# Case Report: Fatal convergence of two confirmed and one radiologically suspected synchronous urological malignancies in a young patient: ultra-late clear cell renal cell carcinoma recurrence, contralateral renal tumor, and metastatic prostate cancer

**DOI:** 10.3389/fonc.2026.1852834

**Published:** 2026-07-09

**Authors:** Youssef Maachi, Houda Fadriq, Hanane El Kacemi, Tayeb Kebdani, Amine Slaoui, Tariq Karmouni, Abdellatif Koutani, Khalid Elhassouni, Khalid Elkhader

**Affiliations:** 1 Department of Urology B, Centre Hospitalier Universitaire (CHU) Ibn Sina, Faculty of Medicine and Pharmacy, Mohamed V University, Rabat, Morocco; 2 Department of Radiotherapy, National Institute of Oncology (INO), Faculty of Medicine and Pharmacy, Mohamed V University, Rabat, Morocco

**Keywords:** hereditary cancer predisposition, metastatic prostate cancer, post-nephrectomy surveillance, synchronous multiple primary malignancies, ultra-late renal cell carcinoma recurrence

## Abstract

**Introduction:**

The simultaneous presentation of multiple primary urological malignancies in a single patient is exceedingly rare and poses significant diagnostic and therapeutic challenges. We report the case of a 48-year-old man presenting two histologically confirmed synchronous urological malignancies and one radiologically suspected contralateral renal mass.

**Case presentation:**

The patient had undergone right radical nephrectomy for pT1 ISUP grade 2 ccRCC fourteen years prior. Surveillance imaging revealed a recurrence at the right nephrectomy bed (12 cm) and an incidentally discovered left renal mass (4 cm). CT imaging additionally identified extensive abdominal dissemination of indeterminate origin. Histological confirmation was obtained for the right renal recurrence by CT-guided biopsy and for the prostate cancer by systematic biopsy; the contralateral left renal mass fulfilled imaging criteria for ccRCC but could not be biopsied prior to the patient’s death, representing a diagnostic limitation acknowledged throughout this report. Biochemical workup simultaneously revealed a markedly elevated PSA of 50 ng/ml. Prostate biopsy confirmed Gleason 9 (5 + 4) adenocarcinoma with bone scintigraphy demonstrating four skeletal metastatic foci. The patient died at home one month after diagnosis, before initiation of treatment.

**Conclusion:**

This case illustrates the exceptional convergence of three synchronous urological conditions—two histologically confirmed and one radiologically suspected—with a fatal outcome, highlighting the importance of extended post-nephrectomy surveillance, integrated PSA monitoring in ccRCC survivors, and early germline evaluation in young patients with multiple urological primaries. synchronous multiple primary malignancies, ultra-late renal cell carcinoma recurrence, metastatic prostate cancer, post-nephrectomy surveillance, hereditary cancer predisposition

## 1 Introduction

Multiple primary malignancies (MPM) occurring within the same organ system represent an uncommon but clinically significant entity. In urology, the coexistence of renal cell carcinoma (RCC) and prostate cancer (PC) has been described in population-based studies, with a modestly increased risk of a second primary malignancy in survivors of either disease (1, 2). The synchronous presentation of three distinct urological conditions, an ultra-late ccRCC recurrence, a contralateral renal tumor, and newly diagnosed high-grade metastatic prostate cancer in a 48-year-old patient constitutes an exceptionally rare scenario with no established management algorithm.

Clear cell RCC accounts for 75%–80% of renal malignancies and is notable for its capacity for ultra-late recurrence, defined as relapse more than ten years after nephrectomy, documented up to three decades post-surgery (3). The additional development of a contralateral renal mass raises the differential between metachronous bilateral primary RCC occurring in 1%–2% of patients after unilateral nephrectomy and late contralateral metastasis (4). *De-novo* metastatic prostate cancer is associated with substantially reduced survival, with median overall survival of 42–60 months with optimal systemic therapy (5), and Gleason 9 disease confers an even more aggressive phenotype.

We present this case to highlight the diagnostic complexity of simultaneous multi-organ urological malignancy and to advocate for structured lifelong surveillance and early germline genetic evaluation in young patients presenting with multiple urological primaries. Although the index nephrectomy was performed 14 years prior, the locoregional recurrence, the contralateral renal mass, and the prostate cancer were all identified concurrently at the time of the current presentation, fulfilling the Warren and Gates criteria for the two histologically confirmed conditions—the right retroperitoneal recurrence and the prostatic adenocarcinoma. The contralateral renal mass, while fulfilling established imaging criteria for ccRCC, could not be biopsied prior to the patient’s death and could not be formally classified as a confirmed third primary. This diagnostic limitation is explicitly acknowledged and represents a central limitation shaping the interpretation of all findings presented herein.

This case report was prepared in accordance with the CARE (CAse REport) guidelines. The completed CARE checklist is provided as Supplementary Material.

## 2 Case presentation

### 2.1 Medical history and oncological background

A 48-year-old male patient presented spontaneously to our urology department with a three-month history of right-sided loin pain localised to the prior nephrectomy bed. His past medical history was notable for a right radical nephrectomy performed 14 years prior for a clear cell renal cell carcinoma staged pT1, ISUP grade 2, with no tumour necrosis and no vascular emboli features conventionally associated with low recurrence risk. The patient had been entirely lost to oncological follow-up following an initial post-operative surveillance period, with no imaging or biochemical monitoring for the preceding several years. No family history of urological or hereditary cancer syndromes was documented, and no germline genetic testing had previously been performed.

### 2.2 Clinical presentation and investigative findings

At presentation, the patient reported progressive right loin pain without haematuria, lower urinary tract symptoms, or constitutional features. He was ambulatory and haemodynamically stable. Laboratory evaluation revealed a serum creatinine of 150 µmol/L consistent with CKD stage G3 (KDIGO) in the context of a solitary functioning left kidney and moderate normocytic anaemia with a haemoglobin of 9.2 g/dl attributable to chronic renal insufficiency and malignant burden. Extended biochemical workup revealed mild hypercalcaemia (2.8 mmol/L); elevated alkaline phosphatase (180 U/L) and lactate dehydrogenase (290 U/L) consistent with significant metastatic burden; and reduced serum albumin (25 g/L) reflecting malignant cachexia and peritoneal carcinomatosis—findings with direct implications for treatment tolerability. Serum PSA was markedly elevated at 50 ng/ml, an incidental but critical finding that prompted immediate systematic urological investigation.

**Figure 1:**
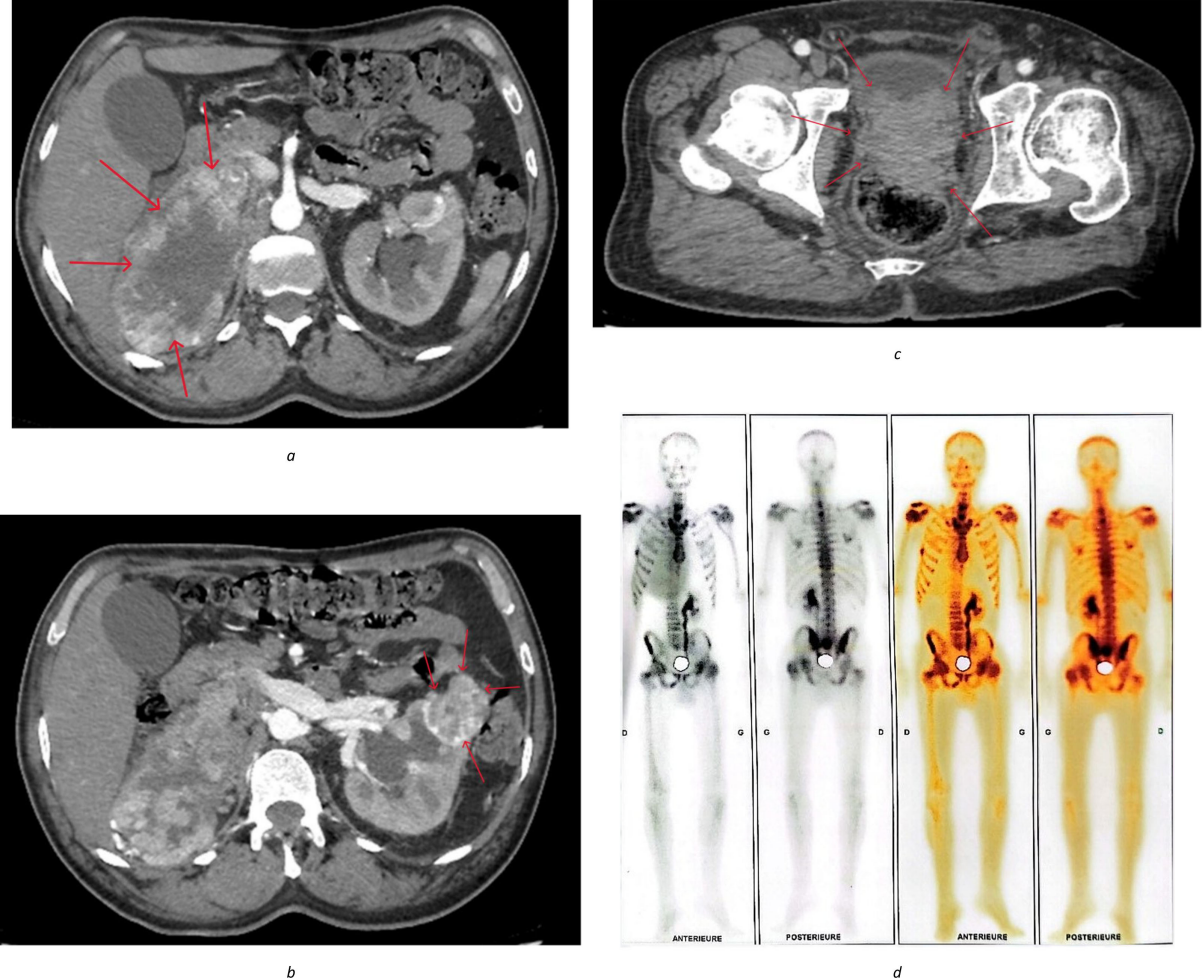
**(a)** Axial contrast-enhanced computed tomography of the abdomen demonstrating a large heterogeneous soft tissue mass measuring 12 cm in the right retroperitoneal region (Red Arrow), occupying the prior nephrectomy bed, consistent with locoregional recurrence of clear cell renal cell carcinoma fourteen years following radical nephrectomy. This finding is consistent with subclinical tumour growth during a prolonged surveillance gap. **(b)** Axial computed tomography in arterial phase demonstrating a 4 cm hypervascular solid mass at the lower pole of the left kidney (red arrow), with significant contrast enhancement and absence of macroscopic fat, consistent with clear cell renal cell carcinoma on imaging criteria. Histological confirmation was not obtained prior to the patient’s death. The imaging characteristics fulfil established radiological criteria for ccRCC; however, the absence of histological confirmation represents a limitation of this case. **(c)** Axial contrast-enhanced computed tomography of the pelvis demonstrating an enlarged, heterogeneous prostate gland with evidence of extracapsular extension (red arrow) and adjacent pelvic lymphadenopathy. These features are consistent with locally advanced prostatic adenocarcinoma at high risk of systemic dissemination. **(d)** Anterior whole-body radionuclide bone scintigraphy demonstrating multiple foci of increased tracer uptake involving the humeral heads, scapulae, right 6th rib arc, cervical and thoracic spine, sacrum, pubis, ilio-pubic ramus, and trochanteric regions, consistent with high-volume osseous metastatic disease. Lesions in the humeral heads, scapulae, and right 6th rib arc are located beyond the vertebral column and pelvis, formally fulfilling CHAARTED high-volume criteria. Degenerative etiology for shoulder girdle uptake cannot be formally excluded without correlative CT or MRI; however, the multiplicity and distribution of lesions in the clinical context of PSA 50 ng/ml and Gleason 9 disease support a metastatic interpretation.

Contrast-enhanced computed tomography (CT) of the thorax, abdomen, and pelvis identified two distinct renal lesions: a 12 cm soft tissue mass at the right nephrectomy bed (**Figure 1a**), consistent with locoregional ccRCC recurrence, and a 4-cm hypervascular solid mass at the lower pole of the left kidney (**Figure 1b**), consistent with renal cell carcinoma on imaging criteria. No thoracic metastases were identified. Pelvic imaging demonstrated an enlarged heterogeneous prostate with extracapsular extension (**Figure 1c**) and pelvic lymph node involvement. Abdominal imaging further revealed bilateral para-aortic lymphadenopathy, perihepatic effusion, and mesenteric infiltration collectively consistent with peritoneal carcinomatosis of indeterminate origin, unconfirmed histologically.

**Figure 2:**
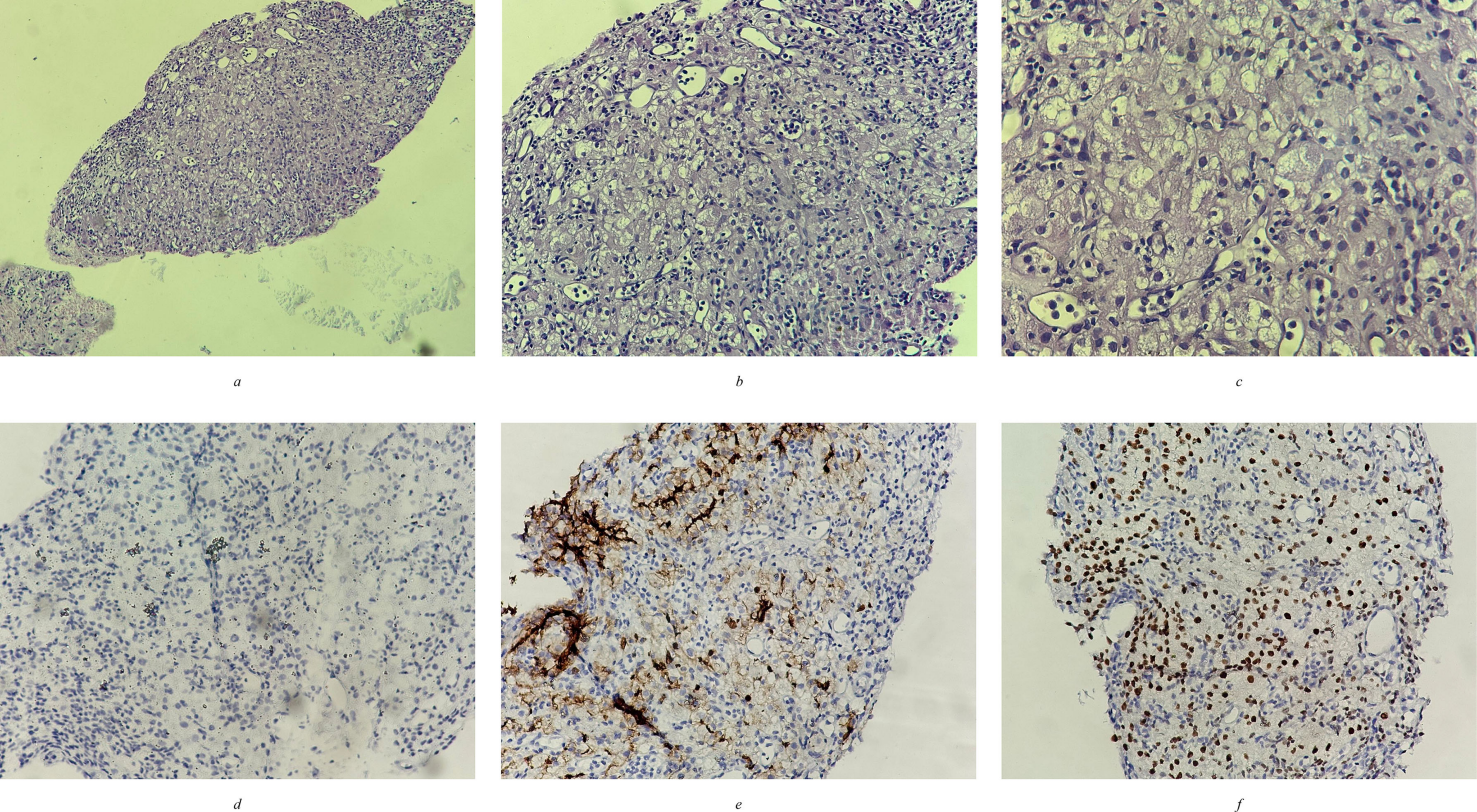
Histopathological analysis of the CT-guided biopsy specimen from the right retroperitoneal mass confirming clear cell renal cell carcinoma recurrence. **(a)** Haematoxylin and eosin staining (×10) demonstrating a proliferation of clear cells arranged in nests separated by thin-walled sinusoidal vasculature, characteristic of clear cell renal cell carcinoma morphology. **(b)** Haematoxylin and eosin staining (×20) showing clear cell cytoplasm with well-defined cell borders and prominent vascular network. **(c)** Haematoxylin and eosin staining (×40) demonstrating nuclear features with round nuclei and nucleoli visible only at high magnification (×400), consistent with ISUP grade 2. **(d)** CK7 immunostaining showing negative expression in tumour cells, consistent with clear cell renal cell carcinoma and effectively excluding urothelial carcinoma. **(e)** CD10 immunostaining demonstrating diffuse positive expression in tumour cells, supporting the diagnosis of clear cell renal cell carcinoma. **(f)** PAX8 immunostaining showing positive nuclear expression in tumour cells, confirming renal tubular origin and supporting the diagnosis of renal cell carcinoma..

CT-guided biopsy of the right retroperitoneal mass confirmed clear cell renal cell carcinoma histologically consistent with recurrence of the original primary tumour. Histopathological analysis of the biopsy specimen is illustrated in (**Figure 2**). Haematoxylin and eosin staining confirmed clear cell morphology with characteristic thin-walled sinusoidal vasculature. Immunohistochemical profiling demonstrated negative CK7 expression, diffuse CD10 positivity, and positive PAX8 nuclear staining, a profile diagnostic of clear cell renal cell carcinoma, highly consistent with locoregional recurrence of the original primary tumour. Systematic transrectal prostate biopsy confirmed Gleason 9 (5 + 4) prostatic adenocarcinoma, Grade Group 5. Systematic biopsy yielded 8 positive cores out of 12 sampled. Clinical staging was cT4N1M1b. Baseline ECOG performance status was 1 at the initial assessment, prior to the rapid functional decline documented in the subsequent weeks.

Radionuclide bone scintigraphy demonstrated multiple foci of increased tracer uptake involving the humeral heads, scapulae, right 6th rib arc, cervical and thoracic spine, sacrum, pubis, ilio-pubic ramus, and trochanteric regions consistent with osseous metastatic disease (**Figure 1d**).

The diagnostic workup addressed three parallel questions. The right retroperitoneal mass was attributed to locoregional ccRCC recurrence based on location and history, confirmed by CT-guided biopsy. The left renal mass raised a differential between metachronous bilateral primary ccRCC and contralateral metastasis; imaging characteristics were consistent with ccRCC on established radiological criteria, but histological confirmation could not be obtained prior to death, leaving this distinction unresolved. The origin of peritoneal dissemination remained undetermined in the absence of peritoneal biopsy, though the radiological pattern was considered most consistent with a prostatic origin. Prognostically, the combination of Gleason 9 disease, PSA 50 ng/ml, high-volume bone metastases, hypoalbuminaemia, and CKD stage G3 placed this patient in an extremely poor prognostic category across all concurrent conditions.

**Table 1** provides a comparative overview of the three synchronous urological conditions, including histological confirmation, staging, grading, and treatment status.

**Table 1:** Summary of the three synchronous urological conditions.

Parameter	Right renal recurrence	Left renal tumor	Prostate cancer
Histology	ccRCC confirmed by CT-guided biopsy—CK7 negative, CD10 positive, PAX8 positive	Suspected ccRCC—not biopsied	Prostatic adenocarcinoma confirmed by systematic biopsy
Size/stage	12-cm locoregional recurrence	4 cm—radiologically suspected RCC; organ-confined appearance on imaging, unconfirmed histologically; benign etiology or metastatic origin cannot be excluded	Metastatic (bone, > 4 foci)—cT4N1M1b
Grade	ISUP grade 2 (original specimen)	Not determined	Gleason 9 (5 + 4) Grade Group 5
Biomarker	N/A	N/A	PSA, 50 ng/ml
Treatment	None initiated—patient died one month after diagnosis	None initiated—patient died one month after diagnosis	None initiated—patient died one month after diagnosis

### 2.3 Clinical course and outcome

Given the extraordinary complexity of the oncological presentation, a multidisciplinary tumour board discussion was planned to determine therapeutic priority, specifically whether immediate systemic treatment for metastatic prostate cancer or surgical management of the renal masses should take precedence.

Therapeutic considerations: No treatment was initiated prior to the patient’s death. The planned approach discussed at the tumor board level would have prioritised systemic therapy for metastatic prostate cancer—androgen deprivation therapy combined with an androgen receptor pathway inhibitor or docetaxel—ahead of any surgical intervention on the renal masses. Management of the ccRCC recurrence and contralateral renal mass would have constituted a second therapeutic sequence, pending response assessment and histological clarification, respectively.

However, before any treatment could be initiated, the patient’s condition deteriorated rapidly. According to family accounts, he experienced progressive functional decline consistent with a performance status of ≥3, rendering him unable to attend scheduled appointments. He was found deceased at home approximately one month after the oncological diagnosis was established. No autopsy was performed, precluding definitive determination of the cause of death.

Multiple competing mechanisms were plausibly contributory, including rapidly progressive high-volume metastatic prostate cancer, acute-on-chronic renal failure in the context of a solitary kidney, hypercalcaemia of malignancy, thromboembolic disease, and peritoneal carcinomatosis-related nutritional failure. No single etiology can be assigned primacy on clinical grounds alone. The extensive peritoneal dissemination identified on imaging is consistent with the rapidity of functional decline, as peritoneal carcinomatosis is independently associated with severe nutritional failure and performance status deterioration regardless of primary origin.

The chronological sequence of oncological events, summarized in **Table 2**, highlights that the entire diagnostic and clinical deterioration trajectory unfolded within a single month following fourteen years of unmonitored interval, a timeline central to understanding the fatal outcome of this case.

**Table 2:** Clinical timeline of oncological events.

Timepoint	Clinical event	Key clinical data
Year 0 (14 years prior)	Right radical nephrectomy for ccRCC	pT1, ISUP grade 2, no necrosis, no vascular emboli. Patient declared disease-free
Years 1–3	Initial post-operative surveillance	Imaging performed, no recurrence detected
Years 4–13	Lost to oncological follow-up	No imaging, no PSA monitoring performed
Current presentation—Week 1	Re-presents with 3-month right loin pain	CT: 12 cm right nephrectomy bed recurrence + 4 cm left renal mass. Hb 9.2 g/dl, Creatinine 150 µmol/L, Albumin 25 g/L, LDH 290 U/L, ALP 180 U/L, Ca 2.8 mmol/L
Weeks 1–2	Biochemical workup	PSA 50 ng/ml
Week 2	Systematic prostate biopsy	Gleason 9 (5 + 4) Grade Group 5 confirmed
Week 2	CT-guided biopsy right mass	ccRCC confirmed histologically
Week 3	Bone scintigraphy	Multiple osseous metastatic foci identified: humeral heads, scapulae, right 6th rib arc, cervical and thoracic spine, sacrum, pubis, ilio-pubic ramus, and trochanteric regions — fulfilling CHAARTED high-volume criteria (>4 foci with at least one beyond the vertebral column and pelvis)
Weeks 3–4	MDT discussion planned	Therapeutic priority: systemic therapy for mHSPC first. No treatment initiated due to rapid deterioration
1 month post-diagnosis	Patient found deceased at home	Cause undetermined. No autopsy performed

## 3 Patient perspective

The patient was informed of his diagnoses by the treating urologist following completion of the diagnostic workup, including bone scintigraphy. Upon learning simultaneously of a recurrent kidney cancer, a contralateral renal mass, and metastatic prostate cancer with bone involvement, he reacted with profound shock. He expressed a wish to receive treatment and was awaiting the multidisciplinary team’s recommendations at the time of his final clinical contact. The family, who provided consent for publication following his death, confirmed that he had gradually lost contact with medical follow-up in the years after his initial surgery, believing himself to be cured. They expressed that the triple oncological diagnosis came as an overwhelming experience and hope that sharing this case may encourage other patients with a history of urological cancer to maintain regular long-term follow-up and prompt clinicians to implement more proactive surveillance strategies.

## 4 Discussion

### 4.1 Ultra-late recurrence of ccRCC: an underrecognized oncological hazard

The occurrence of ccRCC recurrence fourteen years after radical nephrectomy represents a clinically notable ultra-late recurrence. While recurrence beyond ten years after nephrectomy is uncommon, it is documented in the literature and should not be considered exceptionally rare in isolation (3, 6). The distinctive feature of the present case is not the ultra-late recurrence per se, but its convergence with a synchronous contralateral renal mass and high-volume metastatic prostate cancer in a 48-year-old patient. While most renal cell carcinoma relapses occur within 3–5 years post-nephrectomy, a subset of patients develops recurrence beyond the decade mark.

In our patient, the favourable initial pathological profile (pT1 stage, ISUP grade 2, absence of tumour necrosis and vascular emboli) did not preclude late locoregional recurrence, consistent with published data demonstrating that even low-grade ccRCC can harbour dormant clones capable of late progression (3). This observation is particularly instructive, underscoring that no combination of favourable prognostic factors can entirely exclude the risk of ultra-late relapse in ccRCC survivors. The recurrence manifested as right loin pain after a prolonged period of loss to follow-up, and the 12 cm mass identified at the nephrectomy bed reflects subclinical growth during the surveillance gap, a direct and preventable consequence of interrupted long-term imaging follow-up. This case adds to the growing body of evidence suggesting that long-term imaging surveillance beyond conventional follow-up intervals warrants consideration in ccRCC survivors, irrespective of initial pathological stage.

### 4.2 Contralateral renal mass: bilateral ccRCC or late metastasis?

The 4 cm left renal mass discovered incidentally introduces a critical differential diagnosis: metachronous bilateral primary ccRCC versus a contralateral metastasis from the original right-sided tumor. Metachronous bilateral renal cell carcinoma occurs in approximately 1%–2% of patients following unilateral nephrectomy, and its incidence is significantly higher in patients with hereditary RCC syndromes, notably von Hippel-Lindau (VHL) disease, hereditary papillary RCC, and BAP1-associated tumor predisposition syndrome (4, 7). In the context of a 48-year-old patient with multiple synchronous malignancies, hereditary predisposition must be strongly suspected.

Distinguishing a bilateral primary from a metastatic lesion requires biopsy with independent histological and molecular characterization of the contralateral tumor. In our patient, this distinction could not be established prior to death, representing a limitation of the workup. From a clinical standpoint, a left partial nephrectomy with curative intent would have been considered had the lesion proven to be an independent primary, given the solitary kidney context and the patient’s age.

### 4.3 Metastatic prostate cancer: high-grade, high-burden, fatal presentation

The diagnosis of Gleason 9 (5 + 4) metastatic prostate cancer Grade Group 5, the highest-risk histological category alongside two concurrent renal malignancies, represents an extraordinary oncological burden. A PSA of 50 ng/ml combined with four bone metastases on scintigraphy unambiguously classifies this patient within the high-volume metastatic hormone-sensitive prostate cancer (mHSPC) category per CHAARTED and LATITUDE trial definitions (5, 8). Per CHAARTED criteria, high-volume disease requires visceral metastases or four or more bone metastases with at least one beyond the vertebral column and pelvis, a definition fulfilled by our patient’s scintigraphic distribution, which included lesions in the humeral heads, scapulae, and right sixth rib arc—all located beyond the vertebral column and pelvis.

These findings are consistent with the only published retrospective series specifically analyzing synchronous RCC and prostate cancer. Hsu et al. (4) reported on 30 patients with concurrent RCC and PC, demonstrating a significantly higher prevalence of high-risk PC in the synchronous group (57.1% vs. 18.7%, *p* = 0.029), a trend toward more advanced RCC staging (50% vs. 18.7% stage III/IV, *p* = 0.07), and significantly shorter overall survival (mean OS 112.4 vs. 164 months, *p* = 0.041), with all metastatic cases confined to the synchronous subgroup. Our patient Gleason 9, PSA 50 ng/ml, four bone metastases, and concurrent active renal malignancies represent the extreme end of this spectrum, beyond what was observed in that series, and further illustrate the prognostic severity of synchronous urological convergence.

Triplet therapy combining ADT, docetaxel, and an androgen receptor pathway inhibitor represents an emerging standard for fit patients with high-volume metastatic hormone-sensitive prostate cancer (8). However, this patient’s hypoalbuminaemia (25 g/L), CKD stage G3, and rapid functional decline to ECOG ≥3 would likely have precluded treatment intensification, illustrating that trial-based regimens are not universally applicable and that individual patient fitness must guide therapeutic decisions in this setting.

From a differential standpoint, the radiological pattern of pelvic lymphadenopathy, omental infiltration, and perihepatic effusion, while peritoneal dissemination from any of the three malignancies cannot be excluded, is radiographically more consistent with a prostatic origin, though peritoneal disease of indeterminate origin cannot be attributed to any single malignancy without histological confirmation, given the rarity of peritoneal spread in ccRCC and the known capacity of high-grade prostate adenocarcinoma for peritoneal involvement in advanced disease.

### 4.4 Genetic predisposition: an unexplored but critical question

The combination of bilateral renal involvement, synchronous prostate cancer, and onset at age 48 strongly suggests an underlying hereditary cancer predisposition syndrome. Several germline candidates warrant consideration. BRCA2 pathogenic variants are associated with early-onset high-grade prostate cancer (Gleason 8–10) and occasional RCC susceptibility (9, 10). VHL germline mutation, the hallmark of von Hippel-Lindau disease, should be specifically assessed given the bilateral ccRCC presentation before age 60, as the solitary contralateral tumor does not exclude this diagnosis. BAP1 germline mutations are similarly associated with bilateral ccRCC and aggressive phenotypes (7).

Germline evaluation was discussed at the multidisciplinary tumor board; however, the patient’s rapid deterioration and death within one month precluded formal oncogenetic referral. This recommendation carries particular urgency given that the patient died before testing could be completed—first-degree relatives including siblings and children, should be offered germline testing for VHL, BRCA2, and BAP1 variants, as identification of a pathogenic variant would carry direct implications for their surveillance protocols and preventive management. This case reinforces that oncogenetic referral should be initiated at the earliest opportunity in young patients with multiple urological primaries.

### 4.5 Narrative overview of reported cases of triple synchronous urological malignancy

A narrative literature search was conducted in PubMed and Google Scholar using the following terms: “synchronous triple urological malignancy,” “multiple primary urological cancers,” “synchronous renal prostate bladder cancer,” and “triple primary genitourinary malignancy,” covering publications from January 1985 to December 2025 in English and Japanese. Inclusion criteria were (1) at least three synchronous primary urological malignancies in a single patient, (2) published as a case report or case series, and (3) sufficient clinical data to allow comparison. Exclusion criteria were secondary malignancies of non-urological origin and cases where synchronicity could not be established. No PRISMA flowchart was generated, as this review does not meet the formal criteria for a systematic review and is presented as a narrative contextualisation of a rare entity.

To the best of our knowledge, this represents the first narrative compilation of all identifiable published cases of synchronous triple urological malignancy reported in the English and Japanese literature from 1985 to 2025, based on the search strategy described above (**Table 3**). The search was last conducted in December 2025; the possibility that eligible cases published in non-indexed journals or in languages other than English and Japanese were missed cannot be excluded.

**Table 3:** Comparative overview of reported cases of synchronous triple urological malignancy (1985–2025).

No.	Authors (year) journal	Age/country	Triad category	Triple malignancy	Grade/PSA	Histologic confirmation	Metastases	Treatment	Outcome
1	Wentworth et al. Urology, 1985 USA (14)	66 y	Kidney-bladder-prostate	Low-grade RCC left kidney+ TCC bladder gr.2 + Prostate ADC stage D	PSA NR/Acid phosphatase 4.9 U/L (elevated)/ALP 120 U/L	Yes	Bone (multiple foci—presumed prostate origin) + Peritoneal implants and periaortic/pericaval lymphadenopathy—confirmed bladder origin at surgery	Diethylstilbestrol + TURBT + Left nephrectomy; radiation therapy planned but not confirmed as administered	NR
2	Takada et al. Hinyokika Kiyo, 2002 Japan (11)	72 y	Kidney-bladder-prostate	ccRCC left kidney 6×5 cm + Papillary TCC bladder + Moderately diff. Prostate ADC	PSA 20 ng/ml Gleason NR	Yes	None N0M0	Total left nephro- ureterectomy + Hilar dissection + TURBT + Prostate biopsy	NR
3	Tiwari et al. Saudi J Kidney, 2012 India (16)	55 y	Kidney-bladder-prostate	ccRCC left kidney T1 + UC bladder T2 gr.II + Prostate ADC T1 moderately diff.	PSA 12 ng/ml Gleason NR	Yes	None N0M0	TURBT + Nephro-ureterectomy + Cystoprostatectomy + Ileal conduit	No recurrence at 2 years
4	Kurose et al. Urol Case Rep, 2020 Japan (19)	78 y	Kidney-bladder-prostate	ccRCC right kidney pT1a Fuhrman 3 + UC bladder pTa low gr. + Prostate ADC Gleason 3 + 4 = 7	PSA 6.31 ng/ml Gleason 7	Yes	Bone Th3/Th8/L4 RCC origin confirmed	Robotic partial nephrectomy + TURBT + Degarelix + Nivo/Ipi + Denosumab	Alive at 1 year, stable
5	Hassan et al. IJSCR, 2020 Morocco (17)	77 y	Kidney-upper tract-prostate (suspected/partial)	Prostate ADC ISUP 3 + UC left excretory tract 3.5 cm + Right kidney tumor—radiologically suspected, under surveillance, not biopsied	PSA 10.83 ng/ml Gleason 4 + 3 = 7	Partial—prostate ADC and left upper tract UC confirmed; right renal tumour under surveillance only	None—localized	Laparosc. nephro- ureterectomy + Radiotherapy + ADT. Right kidney: Surveillance	Stable at 6 months
6	Deng et al. Anticancer Res, 2022 USA (20)	81 y	Penile-bladder-prostate	Penile SCC + UC bladder pTa low gr. + Prostate ADC Gleason 10 GG5	PSA NR Gleason 10 GG5	Yes	None N0M0	Partial penectomy + TURBT. ADT not pursued (Alzheimer’s)	No recurrence at 3 months
7	Perry et al. Cureus, 2023 USA (15)	57 y	Kidney-bladder-prostate	UCC bladder MIBC pT3a high gr. + RCC chromophobe left kidney pT3 + Prostate ADC Gleason 3 + 4 GG2 (incidental)	PSA not done Gleason 7 GG2	Yes	None N0M0	Cystoprostatectomy + Nephro-ureterectomy in single stage — 2nd case worldwide	No recurrence at 1.5 years
8	Huang et al.* Front Oncol, 2025 China (18)	57 y	Upper urinary tract triple malignancy: renal parenchyma + renal pelvis + ureter — no prostate involvement	Papillary ccRCC T1b + Verrucous carcinoma renal pelvis T1 + UC ureter T2 (same anat. unit)	PSA NR Low/moderate grades	Yes	Adrenal + Retroperitoneal + Peritoneal at 4 months (late metastases)	Laparosc. nephro- ureterectomy + TURBT + Gemcitabine/ Cisplatin	Progression at 9 months
9	Satoh et al. Hinyokika Kiyo, 2003 Japan (12)	82 y	Kidney-bladder-prostate	RCC left kidney + Infiltrating TCC bladder + Well-diff. Prostate ADC	PSA NR	Unclear—abstract only	None reported	Radical left nephrectomy + Cystoprostatectomy + Ileal conduit	NR
10	Funahashi et al. Hinyokika Kiyo, 2007 Japan (13)	74 y	Kidney-upper tract-prostate with unconfirmed bladder involvement	RCC right kidney + UC left renal pelvis + Prostate ADC; bladder involvement noted — independence of bladder lesions from renal pelvic tumour unconfirmed	PSA 3.4 ng/ml	Unclear—bladder lesion independence not established	Local advanced (renal vein)	Right nephrectomy then left nephro-ureterectomy + Cystectomy	NR
11	Terada. Pathol Res Pract. 2010. Japan (21)	73 y	Upper urinary tract triple malignancy	SCC kidney + SCC ureter + Sarcomatoid UC bladder (80% sarcomatoid + 20% high-grade UC)	PSA NR Low-moderate grades	Yes—but clonal independence of ureteral lesion uncertain	None at diagnosis	Cystectomy + left nephro-ureterectomy	Alive at 3 months NED
12	Ogawa et al. BMC Res Notes. 2014. Japan (22)	67 y	Penile-bladder-prostate	Small cell carcinoma bladder pT3aN0M0 + Ductal ADC prostate + Penile SCC pT1N0M0	PSA NR NSE/ProGRP normal	Yes	None N0M0	Laparoscopic radical cystectomy + urethrectomy + partial penectomy + ileal conduit + adjuvant cisplatin/irinotecan	Alive at 6 months NED
13	Ando et al. Int J Urol. 1996. Japan (23)	59 y	Kidney-upper tract-bladder	ccRCC right kidney pT2 grade 2 + TCC left renal pelvis pT3 grade 2-3 + TCC bladder pTa grade 1	PSA NR	Yes	Suspected left parietal bone—not confirmed on CT	Left nephro-ureterectomy + TURBT + surgical enucleation right RCC + chemotherapy	Alive at 21 months; recurrent bladder TCC treated by TURBT
14	Genta & Rosen. Urology. 1994. USA (24)	67 y	Urethral-penile-prostate	TCC anterior urethra (fossa navicularis) high-grade + SCC penile skin microinvasive + Prostate ADC Gleason 2 (low-grade)	PSA NR Gleason 2	Yes	inguinal lymph nodes (TCC origin)	Total penectomy + inguinal lymph node dissection	NR—metastatic TCC in 4/15 inguinal nodes at resection
★	Our Case 2025 Morocco	48 y	Kidney-kidney-prostate (suspected/partial)	ccRCC—ultra-late recurrence 14 yrs (12 cm) + Contralateral de novo radiologically suspected renal mass (4 cm) + Prostate ADC mHSPC	PSA 50 ng/ml Gleason 9 (5 + 4) GG5	Partial—right renal recurrence and prostate adenocarcinoma confirmed; contralateral renal mass radiologically suspected only	>4 bone foci + Peritoneal carcinomatosis Extreme tumor burden	None initiated—MDT discussion held, systemic therapy for mHSPC planned as first priority; patient died before treatment commencement	Death at 1 month post-diagnosis, before any treatment could be commenced

[ccRCC, clear cell renal cell carcinoma; TCC, transitional cell carcinoma (retained for historical cases predating current nomenclature); UC, urothelial carcinoma; ADC, adenocarcinoma; TURBT, transurethral resection of bladder tumor; SCC, squamous cell carcinoma; MIBC, muscle-invasive bladder cancer; ADT, androgen deprivation therapy; NR, not reported; GG, Grade Group; diff., differentiated; nephro-ureterectomy, radical nephroureterectomy; laparosc., laparoscopic; mHSPC, metastatic hormone-sensitive prostate cancer; ★, index case.].

*Huang et al. represents an upper urinary tract triple malignancy without prostate involvement and is anatomically distinct from the kidney-bladder-prostate pattern characterising the majority of reported cases. It is retained in the table for completeness but should not be interpreted as equivalent to the kidney-bladder-prostate triad.

Cases reported in Japanese-language journals were identified via PubMed and characterised based on available English-language abstracts.

Note.Cases identified but excluded: Das & Brosman (25)—predates search period; acknowledged as potential index case of triple synchronous urological malignancy preceding the present compilation. Charles et al. (26)—two histologically distinct tumours within the same kidney plus ipsilateral ureteral and bladder TCC; does not fulfil criterion of three independent organ-based primaries. Gönül et al. (27)—two synchronous renal tumours within the same kidney; does not fulfil criterion of three independent organ-based primaries.

Fourteen previously published cases were identified: three early Japanese series reported in Hinyokika Kiyo (11–13), one American case (14), one additional American case report (15), one Indian case (16), one Moroccan series (17), one Chinese report (18), one Japanese case (19), one American penile-bladder-prostate case (20), one Japanese upper urinary tract case (21), one Japanese penile-bladder-prostate case (22), one Japanese kidney-upper tract-bladder case (23), and one American urethral-penile-prostate case (24). Three cases involved non-renal urological malignancies as primary components of the triad—penile squamous cell carcinoma (20, 22) and anterior urethral transitional cell carcinoma (24)—representing instances of non-renal triple urological convergence.

Across all 15 cases, including the present one, several consistent patterns emerge: a male predominance with a median age of 67 years (range: 48–82), a predominant inclusion of prostate adenocarcinoma as 1 of the 3 concurrent malignancies (11–17, 19, 20, 22, 24), and a broad spectrum of outcomes ranging from no evidence of disease at two years (16) to death within one month (present case).

The majority of previously reported cases presented without distant metastases at diagnosis (11–13, 15–17, 20–24), and surgical resection with curative intent was feasible in most (11–13, 15–17, 19, 21–23). Only two cases presented with bone metastases (14, 19), and in both instances systemic therapy was successfully initiated, a stark contrast to the present case.

Notably, our patient stands as the youngest reported case by a margin of nine years (16), the only case with confirmed high-volume bone metastases fulfilling CHAARTED criteria at presentation, and the only documented fatality occurring before treatment initiation among cases with available outcome data—outcome data were not reported for several earlier cases, and this claim should be interpreted accordingly. Features that collectively distinguish this case within the reported series: youngest patient age (48 years); highest confirmed metastatic burden (high-volume bone metastases fulfilling CHAARTED criteria, PSA 50 ng/mL, Gleason 9); and only documented fatal outcome before treatment commencement among cases with available outcome data.

**Table 3** presents a case-by-case comparative overview of all identified reports, detailing patient demographics, tumour histology, disease staging, metastatic status, therapeutic approach, and clinical outcome for each published case from 1985 to the present.

### 4.6 Clinical lessons and practical recommendations

This case generates four actionable lessons for urological and oncological practice.

First, extended surveillance beyond five years may be considered in selected ccRCC survivors, particularly those with a history of loss to follow-up, as ultra-late recurrence—while uncommon—remains biologically plausible. The present case illustrates that even low-grade tumours are not exempt from this risk and that interruption of follow-up may allow subclinical disease to reach advanced volume before clinical presentation.

Second, age-appropriate PSA screening should not be neglected in ccRCC survivors. In the present case, PSA measurement was the sole mechanism by which a concurrent lethal malignancy was identified, illustrating the clinical value of maintaining standard preventive screening in this population regardless of renal oncological history.

Third, young patients under 55 years presenting with bilateral renal tumours or multiple urological primaries should be considered for germline genetic evaluation, given the implications for clinical management and family cascade testing.

Fourth, multidisciplinary tumour board engagement should be initiated immediately upon recognition of synchronous multi-organ malignancy to determine oncological priority and individualise treatment sequencing.

### 4.7 Study limitations

Several limitations must be acknowledged. The contralateral left renal mass could not be biopsied or surgically characterised prior to death, precluding definitive distinction between a metachronous primary RCC and a contralateral metastasis, a distinction requiring independent histological and molecular analysis, including VHL mutation profiling. No germline genetic testing was performed, leaving a hereditary predisposition to most plausibly von Hippel-Lindau disease or a BRCA2 pathogenic variant unresolved. The absence of a post-mortem examination limits determination of the precise cause of death and precludes any attribution of primacy among the multiple competing terminal mechanisms identified clinically. Prolonged loss to follow-up introduces gaps in longitudinal data, hindering disease trajectory reconstruction. Serum phosphate and Hounsfield unit measurements of the left renal mass were unavailable, limiting complete radiological characterisation. Finally, peritoneal abnormalities were not histologically assessed, preventing definitive attribution to any of the three concurrent malignancies.

## 5 Conclusion

This case illustrates the exceptional convergence of two histologically confirmed and one radiologically suspected synchronous urological malignancies—ultra-late locoregional ccRCC recurrence, a contralateral renal mass, and Gleason 9 metastatic prostate cancer—culminating in a fatal outcome one month after diagnosis before any treatment could be initiated. The clinical lessons drawn—extended post-nephrectomy surveillance, integrated PSA monitoring in ccRCC survivors, and early germline evaluation in young patients with multiple urological primaries—are detailed in Section 4.6 and are reinforced by the fatal outcome of this case.

## Data Availability

The original contributions presented in the study are included in the article/supplementary material. Further inquiries can be directed to the corresponding author.
